# Optimisation of Microwave‐Assisted Extraction and Spray Drying for Chlorophyll Powder Production From *Ceratophyllum submersum* L.

**DOI:** 10.1002/fsn3.72320

**Published:** 2026-09-03

**Authors:** Hoang Thi Ngoc Nhon, Lam Nguyen Dan Khoa, Le Thi Hong Anh

**Affiliations:** ^1^ Ho Chi Minh City University of Industry and Trade Ho Chi Minh City Vietnam

**Keywords:** *Ceratophyllum submersum*, chlorophyll, microwave extraction, natural colorant, RSM, spray drying

## Abstract

This study aimed to optimize chlorophyll extraction from *Ceratophyllum submersum* L. and develop a stable spray‐dried chlorophyll powder. Microwave‐assisted extraction was optimized using Response Surface Methodology (Box–Behnken design) with MgCO_3_ concentration, microwave power, and extraction time as variables. The optimal conditions (0.763% MgCO_3_, 372.1 W, 5.28 min) yielded 0.187 mg/g dry mass, which closely matched the predicted value. Spray drying parameters were optimized at 25% maltodextrin, a feed rate of 6 mL/min, and an inlet temperature of 160°C. SEM revealed spherical particles with typical surface wrinkling; FTIR showed spectral features consistent with the chlorophyll–maltodextrin matrix, without allowing specific differentiation between chlorophyll and its degradation products, while XRD revealed a predominantly amorphous structure. The resulting powder exhibited a low moisture content (3.25%), high solubility (83.72%), and acceptable flowability, with a Carr index of 23.45% and a Hausner ratio of 1.30. The measured microbiological, heavy‐metal, and residual‐solvent levels met the evaluated safety criteria. These findings demonstrate the potential of *C. submersum* as an underutilized source of natural chlorophyll and provide laboratory‐scale processing conditions for further compositional, storage‐stability, application, and scale‐up studies.

## Graphical abstract

An integrated microwave‐assisted extraction (MAE) and spray‐drying process was developed and optimized by response surface methodology (RSM) to produce a stable chlorophyll powder from the underutilized aquatic macrophyte *Ceratophyllum submersum* L. The resulting powder combined high pigment retention (67.81% chlorophyll; 63.74% recovery) with favorable technological properties (83.72% solubility; 3.25% moisture; Carr index 24.45%, Hausner ratio 1.30) and satisfactory microbiological, heavy‐metal, and residual‐solvent safety, supporting its potential as a natural green colorant for food applications.

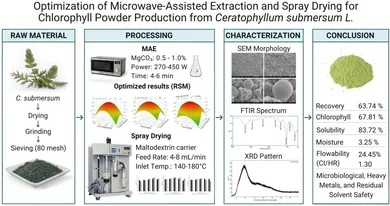

## 1 Introduction

In freshwater ecosystems, aquatic macrophytes play important roles in nutrient cycling, habitat structuring, and eutrophication regulation. The genus *Ceratophyllum*, within the family Ceratophyllaceae in the order Ceratophyllales, comprises submerged, rootless aquatic plants widely distributed across temperate and tropical regions (Bornette and Puijalon 2011). *Ceratophyllum submersum* L. commonly occurs in shallow, nutrient‐rich freshwater bodies and can be distinguished from closely related species by morphological and taxonomic characteristics, particularly fruit morphology and leaf‐branching patterns (Meckoni et al. 2023). Although its rapid biomass accumulation may interfere with aquaculture operations, this species represents a potentially valuable bioresource. Its biomass contains structural carbohydrates, proteins, minerals, and photosynthetic pigments, including chlorophylls a and b (Bornette and Puijalon 2011). The circular bioeconomy can address environmental management and resource efficiency by valuing biomass to produce high‐value products.

Chlorophyll serves as the primary mediator of light energy conversion in photosystems I and II in higher plants, algae, and cyanobacteria. Structurally, chlorophyll consists of a chlorin ring coordinated with a central Mg^2+^ ion and a hydrophobic phytol tail anchoring the molecule within thylakoid membranes. Chlorophyll a and b differ structurally, with a methyl group replaced by a formyl group, resulting in complementary absorption spectra and improved light utilization efficiency (Schwartz and Lorenzo 1990). Beyond its biological function, chlorophyll has attracted increasing attention as a natural food colorant (E140) and bioactive compound due to its antioxidant and health‐promoting properties (Lanfer‐Marquez et al. 2005; Martins et al. 2023). However, it is highly susceptible to heat, light, oxygen, and acidic pH, which promote demetallation to pheophytin and oxidative degradation, thereby limiting stability during processing and storage (Sonar et al. 2019; Hosikian et al. 2010). Optimized extraction and stabilization strategies are therefore essential to preserve pigment integrity and functionality. MgCO_3_ was used as a color stabilizer to minimize acid‐catalyzed chlorophyll demetallation, whereby the central Mg^2+^ is replaced by protons, forming olive‐brown pheophytins. By neutralizing acids released during extraction and maintaining a near‐neutral pH, MgCO_3_ limits pheophytin formation and improves chlorophyll and green‐color retention, consistent with the established pigment‐stabilizing effects of carbonate and metal salts (Molina et al. 2023). Industrial extraction, processing, and storage are confronted with a significant challenge due to this instability. To preserve pigment integrity and functionality, optimized extraction and stabilization strategies are crucial.

Chlorophyll is a magnesium‐porphyrin pigment essential for photosynthesis and widely recognized for its antioxidant, antimutagenic, and potential chemopreventive properties, supporting its application in functional foods and pharmaceuticals. Extraction strategies have predominantly targeted microalgae such as *Spirulina* (*Arthrospira*) and *Chlorella*, as well as leafy vegetables, due to their high pigment content (Hosikian et al. 2010). Food‐grade ethanol extraction remains widely used and is often intensified by ultrasound or enzymatic pretreatment to enhance mass transfer. More recently, supercritical CO_2_ extraction has emerged as a sustainable alternative that can improve pigment recovery while reducing solvent residues and oxidative deterioration (Georgiopoulou et al. 2022; Morcelli et al. 2021). Among the available extraction technologies, microwave‐assisted extraction (MAE) was selected in the present study because its rapid, volumetric heating—generated through direct dipolar and ionic coupling with the solvent—intensifies cell‐wall rupture and mass transfer, thereby shortening extraction time and lowering solvent consumption relative to conventional maceration, ultrasound‐assisted, or enzymatic pre‐treatment (Nonglait and Gokhale 2024). Nevertheless, excessive microwave power or prolonged exposure may accelerate thermal and oxidative chlorophyll degradation. Microwave power and extraction time must therefore be jointly optimized to balance enhanced mass transfer against pigment preservation.

For stabilization and formulation, spray drying is commonly employed to produce chlorophyll powders with improved handling and shelf stability, where process parameters such as inlet temperature, feed rate, and solids content critically govern pigment retention and particle characteristics (Cal and Sollohub 2010). The incorporation of carrier agents such as maltodextrin further raises the glass transition temperature and enhances oxidative stability, thereby minimizing stickiness and pigment loss during drying and storage (Anandharamakrishnan 2015). As a versatile single‐step technique for converting heat‐sensitive liquid feeds into stable, free‐flowing powders, spray drying has proven effective across a wide range of protein‐rich and biomass‐derived materials. For instance, Haas et al. demonstrated that spray drying of whey protein concentrate preserved key bioactive proteins as effectively as freeze drying, while Mansor et al. optimized the operating conditions to recover high‐quality protein powder from rice bran milling by‐products without the use of synthetic drying aids (Haas et al. 2024; Mansor et al. 2022). Collectively, these studies underscore the adaptability of spray drying in retaining the functional and nutritional quality of diverse substrates, supporting its application in the present study.

Recent evidence supports integrating intensified extraction with pigment stabilization to improve chlorophyll recovery. However, extraction conditions must be carefully controlled because temperature, solvent composition, and exposure time strongly affect pigment recovery and stability (Ngcobo et al. 2024). Alkaline salts such as Na_2_CO_3_ can improve chlorophyll recovery and color retention by reducing acid‐induced pheophytinisation (Cao and Dong 2023). Following extraction, encapsulation with maltodextrin and whey protein isolate by spray‐ or freeze‐drying enhances chlorophyll stability against adverse pH, light, and storage conditions (Ledari et al. 2024). Collectively, these findings support the present strategy, which combines controlled MAE, MgCO_3_‐assisted pigment protection, and carrier‐based spray drying to produce a stable chlorophyll powder.

Despite advances in chlorophyll extraction and stabilization from microalgae and terrestrial plants, the potential of *C. submersum* as an alternative pigment source remains insufficiently investigated. To the best of our knowledge, this is the first study to integrate RSM‐optimized MAE, MgCO_3_‐assisted chlorophyll stabilization, and spray drying for powder production. The novelty of the work lies in repurposing a biomass that is normally discarded as an aquaculture nuisance within a circular‐bioeconomy framework; integrating microwave‐assisted extraction with spray‐dried stabilization and optimizing both stages, with Mg^2+^ (MgCO_3_) stabilization incorporated as an explicit extraction variable. In addition, the resulting product was comprehensively characterized in terms of its physicochemical and functional properties, microstructure and solid‐state characteristics using SEM, FTIR, and XRD, as well as food‐safety indicators, including heavy metals, residual solvents, and microbiological quality. Overall, this integrated approach establishes a scientific and technological basis for converting nuisance aquatic biomass into a stable natural green colorant, thereby supporting resource recovery, waste reduction, and the circular bioeconomy in food and related applications.

## 2 Materials and Methods

### 2.1 Materials

Fresh *C. submersum* L. was collected from Dien Chau Ward, Soc Trang Province, Vietnam, and transported to the laboratory on the day of harvest. To eliminate any adherent impurities, the biomass was thoroughly washed with tap water and temporarily stored at −5°C until further processing. The sample was dried in a hot‐air oven at approximately 50°C to constant weight, ground to a fine powder, and passed through an 80‐mesh sieve before analysis. A drying temperature of approximately 50°C was selected to achieve constant weight while minimizing the thermal and oxidative degradation of chlorophyll, as temperatures above 60°C can accelerate pheophytin formation in green tissues (Heaton and Marangoni 1996). The final moisture content of the dried powder was maintained below 10% (w/w). The prepared powder was stored in airtight containers under light‐protected conditions until use.

Ethanol, methanol (Sigma‐Aldrich), and maltodextrin (HiMedia) were obtained from commercial suppliers in Vietnam. Distilled water was used throughout all experimental procedures. All reagents were of analytical grade.

### 2.2 Methods

#### 2.2.1 Optimizing Extraction

The extraction conditions for chlorophyll from *C. submersum* were optimized using Response Surface Methodology (RSM) and a Box–Behnken design (BBD). To determine the main variables that influence chlorophyll recovery, preliminary single‐factor experiments were conducted first. The solvent parameters for aqueous ethanol (80%, v/v, food grade) at a solid‐to‐solvent ratio of 1:30 (w/v) were selected from our previous report (Anh et al. 2026). The experimental design for multivariate optimisation was updated to include the selected variables. The response variable was based on the total chlorophyll content. A second‐order polynomial regression model was used to describe the relationship between independent variables and the response in a general form.

(1) $$Y=\beta_{o}+\sum_{i=1}^{3}\beta_{i}X_{i}+\sum_{i=1}^{3}\beta_{\mathrm{ii}}X_{i}^{2}+\sum_{i=1}^{3}\sum_{j=i+1}^{3}\beta_{\mathrm{ij}}X_{i}X_{j}$$

where *Y* represents the predicted response (total chlorophyll content); *X*_*i*_ and *X*_*j*_ denote the coded levels of the independent variables; *β*₀ is the intercept term; and *β*_*i*_, *β*_*i*__*i*_, and *β*_*i*__*j*_ correspond to the linear, quadratic, and interaction coefficients, respectively. The model‐predicted optimal extraction conditions were experimentally validated. Experimental design, construction, and statistical analyses were performed using JMP software (version 10, SAS Institute Inc., Cary, NC, USA).

To identify the main variables influencing chlorophyll recovery and to define their effective operating ranges, the levels of the independent variables were selected on the basis of preliminary single‐factor experiments together with our previous study on chlorophyll extraction from *C. submersum* (Anh et al. 2026), in which MgCO_3_ concentration, microwave power, and extraction time were found to be the most significant factors governing chlorophyll recovery. Accordingly, three independent variables were investigated at three coded levels (−1, 0, +1): MgCO_3_ concentration X_1_ (0.50, 0.75, 1.00%, w/w), microwave power X_2_ (270, 360, 450 W), and extraction time X_3_ (4, 5, 6 min). A Box–Behnken design was employed to generate 15 experimental runs, including three replicates at the center point to estimate pure error. The complete design matrix, comprising both the coded and actual values together with the corresponding chlorophyll content, is presented in Table 1.

**TABLE 1:** Effects of independent variables on chlorophyll content based on the three‐factor Box–Behnken design.

No.	Coded			Real			Chlorophyll content (mg/gDM)
	X_1_	X_2_	X_3_	MgCO_3_ (%)	Power (W)	Time (min)	
1	−1	−1	0	0.5	270	5	0.142
2	−1	+1	0	0.5	450	5	0.152
3	+1	−1	0	1	270	5	0.141
4	+1	+1	0	1	450	5	0.154
5	0	−1	−1	0.75	270	4	0.14
6	0	−1	+1	0.75	270	6	0.147
7	0	+1	−1	0.75	450	4	0.156
8	0	+1	+1	0.75	450	6	0.164
9	−1	0	−1	0.5	360	4	0.149
10	+1	0	−1	1	360	4	0.147
11	−1	0	+1	0.5	360	6	0.164
12	+1	0	+1	1	360	6	0.169
13	0	0	0	0.75	360	5	0.189
14	0	0	0	0.75	360	5	0.190
15	0	0	0	0.75	360	5	0.192

#### 2.2.2 Spray Drying

The chlorophyll extract was pre‐purified with hexane in a separating funnel; the upper phase was then evaporated to dryness in a vacuum rotary evaporator at 50°C to obtain the crude chlorophyll extract, which was subsequently mixed with maltodextrin at carrier concentrations of 10%–30% (w/v). Spray drying was performed in a laboratory‐scale spray dryer (SD−1000, Eyela, Japan) under controlled conditions. To evaluate the influence of carrier concentration, samples were dried at a fixed inlet air temperature of 160°C and a feed flow rate of 6 mL/min. Subsequently, the effects of inlet air temperature (140°C–180°C) and feed flow rate (4–8 mL/min) were investigated, while other parameters were maintained at the optimal levels determined in the preceding experiments. The obtained powders were collected, packed in polyethene bags, wrapped with aluminium foil to minimize light exposure, and stored at 4°C–5°C until analysis. Powder recovery (%) was calculated as the ratio of collected powder mass to the initial dry solids in the feed solution multiplied by 100. Initial dry solids were determined by oven‐drying the feed to constant weight. Total chlorophyll content was determined by UV–Vis spectrophotometry. The chlorophyll retention was calculated via Equation (2).

(2) $$\text{Chlorophyll retention}\;\left(\%\right)=\frac{C_{p}\times m_{p}}{C_{f}\times V_{f}}\times 100\%$$

where $C_{p}$ is the chlorophyll concentration in the powder (mg/g), $m_{p}$ is the mass of powder recovered (g), $C_{f}$ is the chlorophyll concentration in the spray‐dryer feed (mg/mL), and $V_{f}$ is the volume of feed processed (mL). This mass‐balance calculation accounts for both pigment concentration and powder recovery.

#### 2.2.3 Structure Characterization

##### 2.2.3.1 Scanning Electron Microscopy (SEM)

The morphology of spray‐dried chlorophyll powder was examined by scanning electron microscopy (SEM). Particle morphology, surface characteristics, and the presence of impurities were qualitatively evaluated (Echlin 2009).

##### 2.2.3.2 Fourier Transform Infrared Spectroscopy (FT‐IR)

FT‐IR analysis was performed using an FT/IR‐6100 spectrometer. Samples were prepared using the KBr pellet method, where 2.5 mg of chlorophyll powder was mixed with 50 mg of dry KBr and compressed into a transparent pellet prior to analysis. Spectra were recorded over 4000–400 cm^−1^ at a resolution of 4 cm^−1^ with 32 co‐added scans, using Spectra Manager II software (JASCO). Characteristic absorption bands corresponding to ester C=O (1700–1650 cm^−1^), conjugated C=C of the porphyrin ring (1600–1500 cm^−1^), and C–O–C stretching (1100–1000 cm^−1^) were identified (Smith 2011).

##### 2.2.3.3 X‐Ray Diffraction (XRD)

The crystalline properties of chlorophyll powder were determined using X‐ray diffraction (XRD). Diffraction patterns were collected over a 2θ range of 5°–80° with a step size of 0.02° and a scanning rate of 2°/min (Pecharsky and Zavalij 2009).

#### 2.2.4 Quality Characterization

The microbiological quality of the spray‐dried chlorophyll powder was assessed by two analyses: the total aerobic mesophilic count, determined by the pour‐plate method on plate count agar incubated at 30°C for 72 h and the total yeast and mold count on Dichloran Rose Bengal Chloramphenicol (DRBC) agar incubated at 25°C for 5–7 days, following the guidelines of the International Commission on Microbiological Specifications for Foods (ICMSF 2011). Results were expressed as colony‐forming units per gram (CFU/g). Excessive mold growth may indicate potential spoilage and risk of mycotoxin contamination, affecting food safety.

#### 2.2.5 Physicochemical Characterization

Moisture Content: Moisture content was determined by the oven‐drying method at 105°C to constant weight, recognized as a reference thermal gravimetric method for dried food materials.

##### 2.2.5.1 Bulk and Tapped Density

Bulk density was determined by gently filling 2 g of powder into a 10 mL graduated cylinder and recording the volume (Nunes and Freitas 2013). Tapped density was measured after mechanically tapping the cylinder (approximately 120 taps) following the description of Ozdikicierler et al. (2014).

Carr Index (CI) and Hausner Ratio (HR) were calculated to evaluate flowability:

(3) $$\mathrm{CI}\;\left(\%\right)=\frac{\text{Tapped density}-\text{Bulk density}\;}{\text{Tapped density}\;}\times 100$$

(4) $$\mathrm{HR}=\frac{\text{Tapped density}}{\text{Bulk density}}$$

Flowability classification was interpreted according to the standard ranges of Hausner (1967).

##### 2.2.5.2 Particle Density and Porosity

Particle density was measured using the liquid displacement method with petroleum ether (Jinapong et al. 2008). Porosity (%) was calculated from bulk and particle densities.

Solubility was determined according to the method of Frank Cone and Ashworth (1947). One gram of powder was dispersed in 100 mL of distilled water, stirred (500 rpm, 30 min), centrifuged (3000 rpm, 5 min), and the supernatant was dried at 105°C. Solubility (%) was calculated based on dissolved solids (Frank Cone and Ashworth 1947).

Heavy metals, including lead (Pb), cadmium (Cd), arsenic (As), and mercury (Hg), were quantified by inductively coupled plasma mass spectrometry (ICP–MS) after microwave‐assisted acid digestion (MARS 6), following AOAC Official Method 2015.01. Briefly, approximately 0.25 g of sample was digested with 65% HNO_3_ and 30% H_2_O_2_ in a MARS 6 microwave digestion system (CEM Corporation, Matthews, NC, USA). The digestion temperature was ramped to 180°C–200°C and held for 20 min. After cooling, the digest was quantitatively transferred and made up to 25 mL with ultrapure water prior to analysis on an Agilent 7900 ICP–MS (Agilent Technologies, Santa Clara, CA, USA). Concentrations were determined against element‐specific external calibration curves. Method validation and quality control, including calibration blanks, reagent blanks, certified reference materials (CRMs), and spike‐recovery tests, were conducted by the accredited analytical laboratory, and the method‐specific limits of detection (LOD) and quantification (LOQ) for each element were obtained from the laboratory's validation records.

Metal concentrations were calculated as follows:

(5) $$\text{Metal}\;\left(\mathrm{mg}/\mathrm{kg}\right)=\frac{C\times V}{m}$$

where C is the measured concentration (μg/L), V is the final volume (L), and m is the sample mass (g).

Quality control included blanks, spike recovery (80–120%), certified reference materials, and triplicate analysis (RSD ≤ 10%). Results were compared with the permissible limits specified in the Vietnamese standard QCVN 4–10:2010/BYT (Pb ≤ 5 mg/kg; As ≤ 3 mg/kg), and the obtained values were also consistent with the limits established by the European Commission Regulation (EC) No 1881/2006.

#### 2.2.6 Residual Solvent Analysis

Residual solvent (n‐hexane) was determined using headspace gas chromatography–mass spectrometry (HS‐GC–MS) following FAO/JECFA (2014) guidelines. Approximately 0.5–1.0 g of sample was placed in a 20 mL headspace vial and analyzed using a GC–MS system equipped with a DB‐624 column (30 m × 0.32 mm × 1.8 μm). Headspace conditions: 90°C incubation for 20 min; injection volume 1 mL. GC oven temperature was programmed from 40°C (5 min) to 200°C at 10°C/min. Detection was performed by electron impact ionization (70 eV), monitoring characteristic ions (e.g., m/z 57 and 71 for hexane). Quantification was performed using an internal standard calibration curve (*R* ^2^ > 0.995). Quality control followed AOAC guidelines, including blank analysis, spike recovery (80%–120%), and repeatability (RSD ≤ 15%). The LOD for residual n‐hexane was 10.0 mg/kg. Residual hexane content was evaluated according to QCVN 4–10:2010/BYT (≤ 50 mg/kg).

#### 2.2.7 Chlorophyll Content Determination

Chlorophyll content was determined using the spectrophotometric method based on its characteristic absorption maxima in the red region of the visible spectrum. Chlorophyll pigments exhibit maximum absorbance at 645 and 663 nm. The absorbance values at these wavelengths are proportional to the concentration of chlorophyll present in the extract. Quantification was performed by measuring absorbance at 645 nm (A_645_) and 663 nm (A_663_) using a UV–Vis spectrophotometer, followed by calculation according to standard equations (Rajalakshmi and Banu 2015).

Chlorophyll concentrations in the extract (μg/mL) were calculated as follows:

(6) $$\text{Total chlorophyll}\;\left(\mathrm{\mu g}/\mathrm{mL}\right)=20.2\times A_{645}+8.02\times A_{663}$$

(7) $$\text{Chlorophyll}\;a\;\left(\mathrm{\mu g}/\mathrm{mL}\right)=12.7\times A_{663}–2.69\times A_{645}$$

(8) $$\text{Chlorophyll}\;b\;\left(\mathrm{\mu g}/\mathrm{mL}\right)=22.9\times A_{645}–4.68\times A_{663}$$

The chlorophyll content was then expressed on a dry weight basis, mg/g dry mass (mg/gDM), using the following conversion formula:

(9) $$\text{Chlorophyll}\;\left(\mathrm{mg}/\mathrm{gDM}\right)=\frac{C\;\left(\mathrm{\mu g}/\mathrm{mL}\right)\times V\;\left(\mathrm{mL}\right)}{1.000\times m\;\left(g\right)}$$

where C is the chlorophyll concentration in the extract (μg/mL), V is the total volume of the extract (mL), m is the initial dry weight of the sample (g), and 1000 is the conversion factor from micrograms to milligrams.

### 2.3 Statistical Analysis

All experiments were conducted in triplicate, and the results were expressed as mean ± standard deviation (SD). Experimental data were initially organized and processed using Microsoft Excel. Statistical analysis was performed by analysis of variance (ANOVA) using Minitab to determine significant differences among treatments. Process optimisation was carried out using JMP. Differences were considered statistically significant at *p* < 0.05.

## 3 Results and Discussion

### 3.1 Optimizing Chlorophyll Extraction

Response Surface Methodology (RSM) was employed to optimize chlorophyll yield obtained through microwave‐assisted extraction. The Box–Behnken model was used to construct a three‐factor, three‐level experimental design to evaluate the effects of the independent variables on chlorophyll content. Three independent variables, MgCO_3_ concentration (X_1_, 0.5%–1.0%), microwave power (X_2_, 270–450 W), and extraction time (X_3_, 4–6 min) were included in the experimental matrix. Each factor was examined at three coding levels (−1, 0, +1). Y, mg/gDM was the chlorophyll content that was the response variable. A total of 15 experimental runs were generated, including three replicates at the center point to estimate pure error and assess model adequacy. The experimental design matrix, including the coded and actual levels of the variables, is presented in Table 1.

The extraction conditions for chlorophyll were optimized via RSM‐assisted microwave‐assisted extraction. Following a three‐factor Box–Behnken design (BBD), the experimental design was constructed. MgCO_3_ concentration (X_1_) was 0.5%–1%, w/w, microwave power (X_2_) was 270–450 W, and extraction time (X_3_) was 4–6 min. Each factor was evaluated at three coded levels (−1, 0, and +1). Model adequacy was evaluated using the coefficient of determination (*R* ^2^), adjusted *R* ^2^, and predictive relevance (*Q* ^2^). In RSM applications for food processing, *R* ^2^ values that are between 0.80 and 0.90 are commonly regarded as adequate, while *Q* ^2^ values exceeding 0.50 indicate good predictive ability (Jiménez‐Contreras et al. 2008). The regression model in this study showed a high fit, with *R* ^2^ = 0.9855 and adjusted *R* ^2^ = 0.9593, which indicates excellent agreement between experimental and predicted values. The *R* ^2^ model was not overfitted and adequately describes the experimental variability, as evidenced by the relatively small difference between *R* ^2^ and adjusted *R* ^2^. Analysis of variance (ANOVA) was used to assess the statistical significance of the model and its coefficients. It was determined that independent variables with *p*‐values below 0.05 were statistically significant. The significant regression coefficients demonstrated that the model was well‐defined and capable of predicting and optimizing chlorophyll extraction during microwave treatment. The estimated regression coefficients are displayed in Table 2.

**TABLE 2:** Effects of independent variables on chlorophyll content.

Chlorophyll content	Coefficient	Standard error	*t*‐value	*p*	Term
Intercept	0.1903333	0.002813	83.38	< 0.0001*	Intercept
X_1_	0.0005	0.001398	0.36	0.7352	MgCO_3_
X_2_	0.007	0.001398	5.01	0.0041*	Power
X_3_	0.00750	0.001398	4.65	0.0056*	Time
X_1_ × X_2_	0.00075	0.001977	0.38	0.7200	MgCO_3_ × Power
X_1_ × X_3_	0.00025	0.001977	0.89	0.4166	MgCO_3_ × Time
X_2_ × X_3_	0.00075	0.001977	0.13	0.9043	Power × Time
X_1_^2^	−0.018792	0.002058	−9.13	0.0003*	MgCO_3_ × MgCO_3_
X_2_^2^	−0.024292	0.002058	−11.81	< 0.0001*	Power × Power
X_3_^2^	−0.014292	0.002058	−6.95	0.0010*	Time × Time

*Note:* *Indicate statistically significant terms at *p* < 0.05.

**FIGURE 1:**
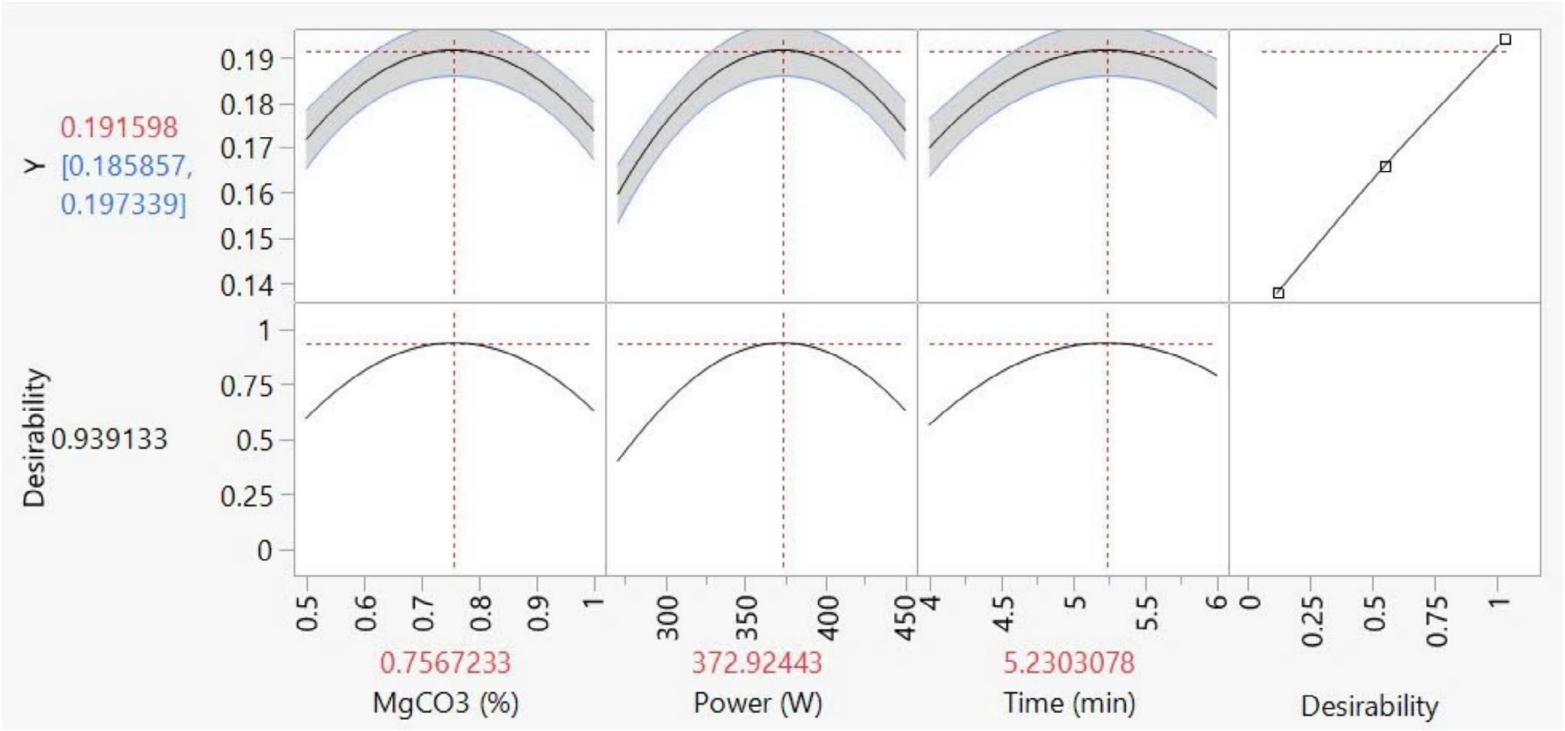
Predictive model for chlorophyll content.

**FIGURE 2:**
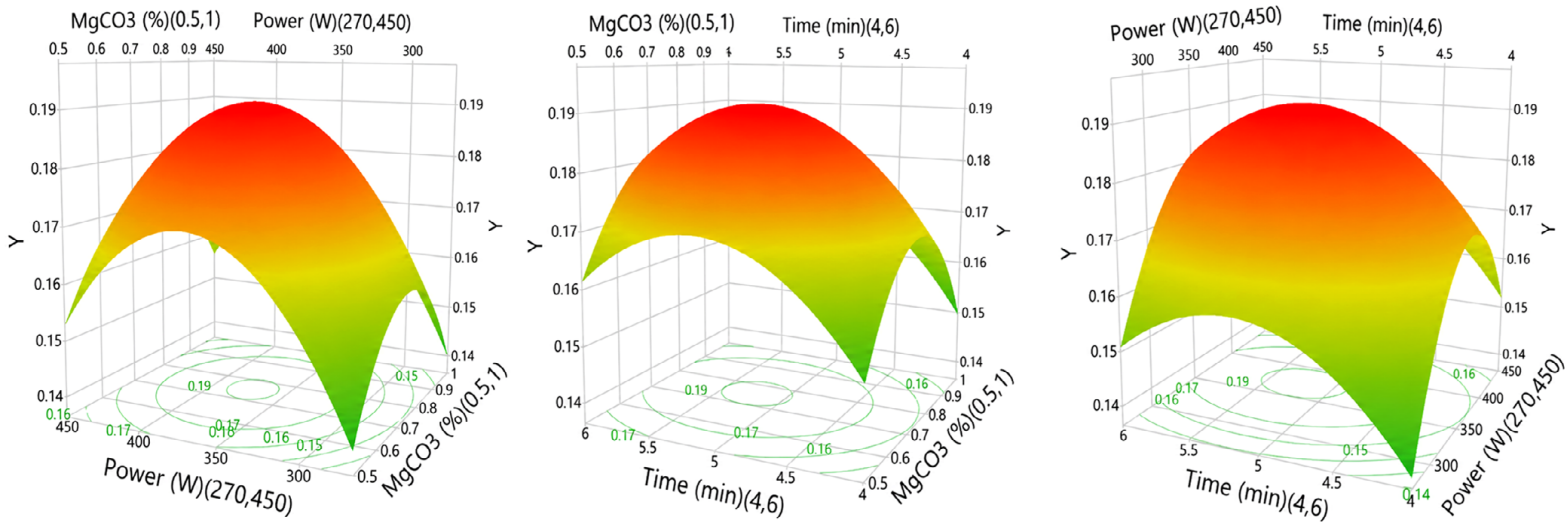
Response surface plots showing the combined effects of MgCO_3_ (*X1*), power (*X2*), and time (*X3*) on chlorophyll content.

At a significance level of *p* < 0.05, the statistically significant coefficients (Table 2) were incorporated into the reduced quadratic regression model as follows:

$$Y=0.1903+0.0070X_{2}+0.0075X_{3}–0.0188{X_{1}}^{2}–0.0243{X_{2}}^{2}–0.0143{X_{3}}^{2}.$$

where Y represents chlorophyll yield, and X_1_, X_2_, and X_3_ are codes for MgCO_3_ concentration, microwave power, and extraction time, respectively. Chlorophyll yield was significantly affected by both microwave power (X_2_) and extraction time (X_3_) in the regression equation, with negative quadratic terms indicating curvature and an optimal region within the investigated ranges. MgCO_3_ concentration exerted a non‐linear effect on chlorophyll recovery: although its linear term was not statistically significant (*p* = 0.7352), the significant negative quadratic term (*p* = 0.0003) indicated the existence of an optimum concentration within the range studied. This can be attributed to the stabilizing role of MgCO_3_, which supplies Mg^2+^ and buffers the extraction medium to limit acid‐induced demetallation, whereas an excessive concentration becomes counter‐productive, giving rise to the observed optimum. The regression model was found to be statistically significant in an analysis of variance (ANOVA) performed with JMP software, supporting its suitability for predicting chlorophyll yield and identifying optimal extraction conditions. The maximum predicted chlorophyll yield was achieved at MgCO_3_ 0.763%, microwave power 372.1 W, and extraction time 5.28 min. MgCO_3_ may play a potential role in stabilizing chlorophyll by providing Mg^2+^, thereby mitigating chlorophyll degradation caused by Mg^2+^ loss during sample processing and extraction. Within the study range, increasing microwave power led to greater heat generation and cell disruption, resulting in the release of chlorophyll into the extraction solvent. Similarly, an optimal extraction time promoted pigment diffusion and mass transfer while avoiding excessive exposure that could accelerate chlorophyll thermal degradation during prolonged microwave heating. Figures 1 and 2 present the interactions between the independent variables (X_1_, X_2_, and X_3_) and the model‐predicted response surfaces.

To validate the model's reliability, confirmatory experiments were conducted at the predicted optimal conditions (*n* = 3). The experimental results showed that the chlorophyll content was 0.187 mg/gDM, with a deviation around 2.5% from the predicted value and well within the acceptable error range (< 5%). These findings demonstrate strong agreement between the predicted and experimental results, confirming the adequacy of the second‐order polynomial model. The strong predictive performance further indicates the model's practical applicability for estimating and optimizing chlorophyll extraction under microwave‐assisted conditions. The positive effect of microwave power can be attributed to enhanced cell wall disruption and improved mass transfer resulting from rapid dielectric heating, which facilitates chlorophyll release into the solvent (Chan et al. 2011). However, excessive power may promote pigment degradation. Similarly, extraction time improves yield up to an optimum threshold, beyond which prolonged exposure to thermal energy accelerates chlorophyll degradation and pheophytin formation. The inclusion of MgCO_3_ contributes to chlorophyll stabilization by supplying Mg^2+^ ions, which may compensate for Mg^2+^ loss during extraction and reduce acid‐induced demetallation. This mechanism helps preserve chlorophyll structure under microwave conditions (Heaton and Marangoni 1996). Numerical optimisation predicted a maximum chlorophyll content at a MgCO_3_ concentration of 0.763%, a microwave power of 372.1 W, and an extraction time of 5.28 min. Under these conditions, the predicted chlorophyll yield reached its maximum value. Model validation experiments (*n* = 3) conducted under optimal conditions yielded 0.187 mg/gDM, showing only 0.9% deviation from the predicted value and remaining within the acceptable error margin (< 5%). This strong agreement confirms the robustness and predictive reliability of the quadratic regression model.

### 3.2 Spray Drying

**FIGURE 3:**
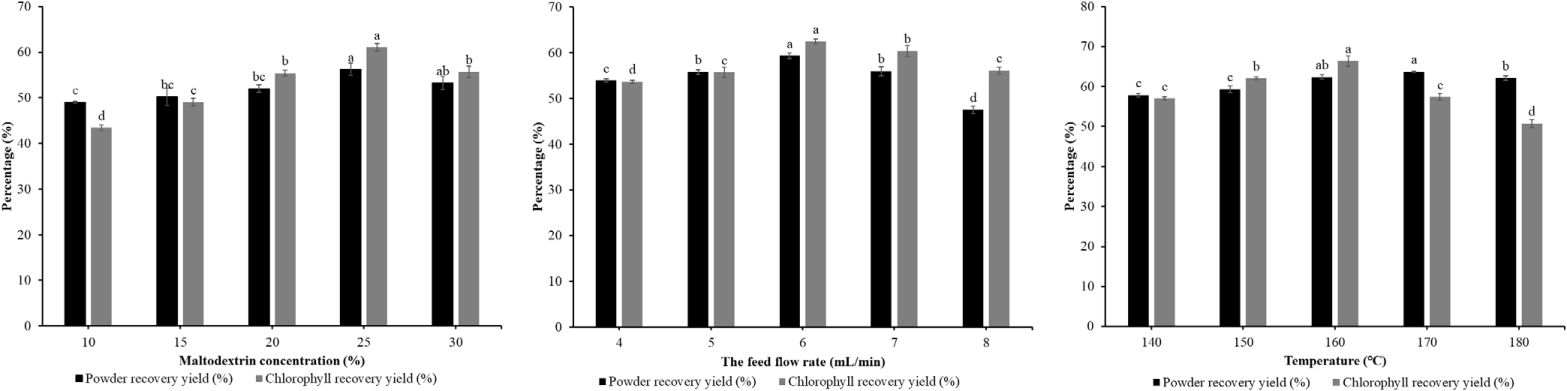
Effects of maltodextrin concentration (A), feed flow rate (B), and inlet air temperature (C) on recovery of chlorophyll powder.

The effects of carrier concentration, feed flow rate, and inlet air temperature on the recovery of chlorophyll powder by spray drying are illustrated in Figure 3.

#### 3.2.1 Effects of Maltodextrin Concentration

A low‐dextrose‐equivalent maltodextrin (DE 10–15) was used in this experiment, as low‐DE grades provide a higher glass‐transition temperature and better film‐forming and barrier properties for pigment encapsulation. The carrier concentration range of 10%–30% (w/v) was established from preliminary single‐factor trials that bracketed the practical spray‐drying window. The influence of maltodextrin concentration (10%–30%, w/v) on powder recovery yield (%) and chlorophyll retention (%) was evaluated at a fixed feed flow rate (6 mL/min) and inlet air temperature (160°C). As illustrated in Figure 3A, increasing the maltodextrin concentration from 10% to 25% resulted in a progressive increase in both powder recovery and chlorophyll retention, reaching maximum values of 56.26% and 61.06%, respectively, at 25% carrier concentration. However, further increasing maltodextrin to 30% led to a decline in both responses. The observed trend can be explained by maltodextrin's physicochemical role as a wall material during spray drying. Maltodextrin was selected as the carrier because it is a food‐grade, cost‐effective, readily available, and organoleptically neutral material with high water solubility. Its low viscosity at high solids content facilitates feed atomisation, while its relatively high glass‐transition temperature reduces particle stickiness and wall deposition during spray drying. Maltodextrin can also provide an oxygen barrier that improves pigment stability (Xiao et al. 2022). Relative to gum arabic it is cheaper and more supply‐stable, and relative to whey protein it avoids allergenicity and Maillard‐type browning that could compromise the green colouration; as the most extensively validated carrier for spray‐dried pigments, it is an appropriate choice for a first optimisation of this novel pigment source (Delaporte et al. 2024). At low solid contents (10%–15%), insufficient carrier concentration results in low feed viscosity and delayed crust formation during droplet drying. Rapid moisture evaporation exposes the chlorophyll‐rich core directly to thermal and oxidative stress, promoting particle collapse, surface stickiness, and adhesion to the drying chamber wall, thereby reducing actual recovery yield. These findings are consistent with the mechanism of particle formation described for spray‐dried bioactive compounds, where inadequate wall material compromises encapsulation efficiency and powder recovery (Gharsallaoui et al. 2007). At intermediate concentrations (20%–25%), the total solids content becomes sufficient to promote rapid glass transition and early film formation at the droplet surface. This enhances structural integrity, reduces stickiness, and protects chlorophyll against thermal degradation and oxidation, resulting in improved retention and recovery. Maltodextrin is widely reported to reduce hygroscopicity and wall deposition due to its relatively high glass transition temperature (Tg), thereby improving process efficiency (Tonon et al. 2008). Conversely, at 30% maltodextrin, excessive viscosity limits internal moisture diffusion and disrupts heat–mass transfer balance. Additionally, the relative proportion of chlorophyll per unit dry solid decreases, which may reduce apparent pigment recovery. This observed trend is consistent with the previous finding that the microencapsulation of chlorophyll as a core material using spray‐drying technology (Rajabi et al. 2023). These results confirm the presence of an optimal maltodextrin threshold for chlorophyll spray drying, beyond which mass transfer constraints and dilution effects outweigh encapsulation benefits.

#### 3.2.2 Effects of Feed Flow Rate

The effect of feed flow rate (4–8 mL/min) was investigated at 25% maltodextrin and 160°C inlet temperature (Figure 3B). Powder recovery and chlorophyll retention increased from 4 to 6 mL/min, reaching maximum values of 59.17% and 65.70%, respectively, at 6 mL/min. A further increase to 7–8 mL/min caused a significant reduction in both parameters. Feed flow rate directly influences droplet residence time and moisture removal kinetics. At low flow rates (4–5 mL/min), droplets remain longer in the drying chamber, potentially leading to overheating, particle shrinkage, and increased wall deposition. Limited feed input also reduces atomisation efficiency, negatively affecting yield. At 6 mL/min, a balance was achieved between evaporation rate and droplet residence time, allowing adequate crust formation while minimizing thermal degradation of chlorophyll. This balance optimizes encapsulation efficiency and reduces stickiness. At higher flow rates (7–8 mL/min), the evaporation capacity of hot air becomes insufficient relative to feed input, leading to incomplete drying and higher particle moisture content. Semi‐dried particles exhibit increased adhesion to chamber walls and cyclone surfaces, decreasing powder recovery. Similar behavior was reported by Santhalakshmy et al., who demonstrated that excessive feed rates in spray drying of natural pigments reduce recovery efficiency due to inadequate moisture evaporation (Santhalakshmy et al. 2015).

#### 3.2.3 Effects of Inlet Air Temperature

The inlet‐air temperature range of 140°C–180°C was investigated to determine the optimal balance between moisture removal and chlorophyll retention (Figure 3C). Evaporative cooling limits droplet heating during the early drying stage, but its effect diminishes as the particles lose moisture, increasing pigment degradation at higher temperatures; conversely, lower temperatures may reduce drying efficiency and increase wall deposition (Ledari et al. 2024; Díaz‐Montes 2025). Increasing the temperature from 140°C to 160°C significantly enhanced powder recovery (58.3% to 62.39%) and chlorophyll retention (57.02% to 67.81%). At 170°C, powder recovery slightly increased (63.74%), but chlorophyll retention decreased markedly. At 180°C, both responses declined substantially. Inlet air temperature governs the rate of moisture evaporation and the dynamics of crust formation. At lower temperatures (140°C–150°C), insufficient thermal driving force results in slower water removal, prolonged droplet wetness, and increased wall deposition. At 160°C, the high temperature gradient accelerates surface drying, promoting rapid crust formation and reducing particle stickiness. This improves powder separation efficiency and protects chlorophyll within a stabilized matrix. At 180°C, rapid surface dehydration may create structural stress and microcracking, increasing oxygen exposure and accelerating pigment degradation. Similar observations were reported by Konar et al. in spray‐dried microalgae powders, where encapsulation efficiency increased up to an optimal temperature but declined beyond the thermal stability threshold of chlorophyll (Konar et al. 2022). Therefore, the data indicate an optimal temperature window (~160°C) for spray‐drying chlorophyll with maltodextrin. Below this range, incomplete drying reduces recovery; above it, thermal degradation dominates. The findings of this study were comparable to those reported for spray‐dried, chlorophyll‐rich materials formulated with maltodextrin. Powder recovery was lower than the 72.98% reported for a chlorophyll‐rich *Chaetomorpha aerea* extract spray‐dried with 25% maltodextrin at 160°C (Hoang et al. 2025), but higher than the 58.55% obtained for a maltodextrin‐containing *Ulva* extract (Ćujić Nikolić et al. 2025). Ledari et al. reported approximately 90% encapsulation efficiency for chlorophyll encapsulated in a maltodextrin–whey protein isolate matrix, with subsequent chlorophyll retention ranging from 41.5% to 94.0% under different pH conditions (Ledari et al. 2024). These values, however, are not directly equivalent to the feed‐to‐powder mass‐balance retention determined in the study. Konar et al. showed that inlet‐air temperature and the relative proportions of maltodextrin and *Chlorella vulgaris* biomass affected the pigment content and physicochemical properties of the resulting powders (Konar et al. 2022), whereas Femat‐Castañeda et al. found that increasing inlet‐air temperature and carrier concentration reduced the Zn^2+^–chlorophyll content of spray‐dried powders prepared with maltodextrin or agave fructans (Femat‐Castañeda et al. 2019). Collectively, these findings demonstrate that pigment preservation and powder recovery depend strongly on the carrier composition, carrier concentration, feed characteristics, and drying conditions.

### 3.3 Chlorophyll Powder Characterization

#### 3.3.1 Morphological Characterization by SEM

**FIGURE 4:**
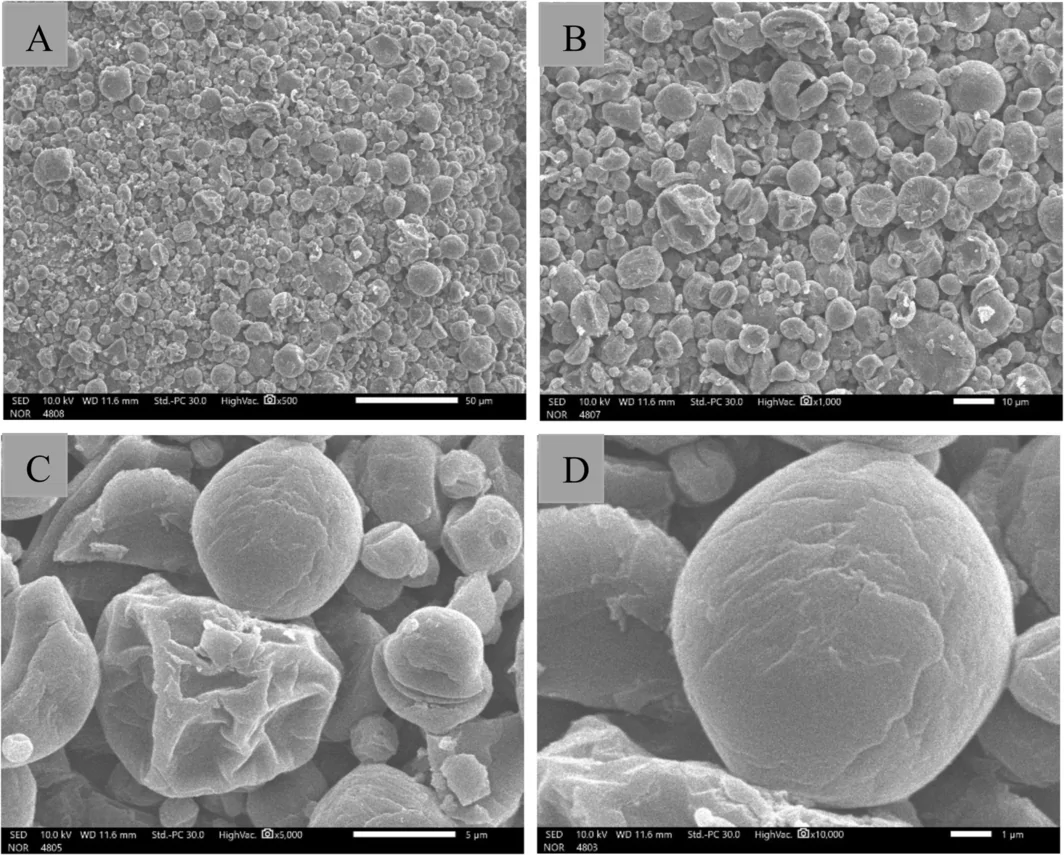
Scanning electron microscopy (SEM) images of spray‐dried chlorophyll powder at magnifications of 500× (A), 1000× (B), 5000× (C), and 10,000× (D).

The microstructural morphology of spray‐dried chlorophyll powder was examined by scanning electron microscopy (SEM) to evaluate particle integrity, surface deformation, and potential inorganic impurities. Representative micrographs at magnifications of 500×, 1000×, 5000×, and 10,000× are presented in Figure 4.

At 500×, most particles exhibited a near‐spherical geometry with slight surface wrinkling and minimal deformation, indicating relatively uniform droplet formation during atomisation. At 1000×, partially flattened particles and curved edges became evident, suggesting localized shell shrinkage. Increasing the magnification to 5000× revealed pronounced surface depressions and wrinkles, consistent with internal moisture diffusion and prolonged solvent evaporation after the formation of a rigid outer crust. At 10000×, the particle surfaces remained continuous without visible cracks, perforations, or crystalline protrusions, indicating complete drying and the absence of structural collapse. This deformation pattern is characteristic of spray‐drying systems containing thermosensitive pigments. Rapid initial solvent evaporation induces the formation of a rigid outer shell, while the still‐moist core continues to contract, leading to surface shrinkage and wrinkle formation. The heterogeneity in evaporation kinetics among droplets likely resulted in two particle populations: (i) smooth, spherical particles with thicker shells, and (ii) concave, wrinkled particles with thinner walls. From a functional perspective, wrinkled particles provide increased surface area, which enhances dispersibility and rehydration capacity; however, the presence of microcapillaries may facilitate moisture uptake and oxygen diffusion, thereby increasing the susceptibility of chlorophyll to oxidative degradation and color instability during storage. In contrast, smooth, compact particles generally exhibit improved mechanical strength and color stability but may dissolve more slowly due to reduced initial water penetration. These observations are consistent with previous findings on spray‐dried chlorophyll encapsulated with maltodextrin, where surface indentation and wrinkling were attributed to rapid evaporation and the brittle nature of the carbohydrate matrix (Kang et al. 2019).

#### 3.3.2 Structural Analysis by FTIR

**FIGURE 5:**
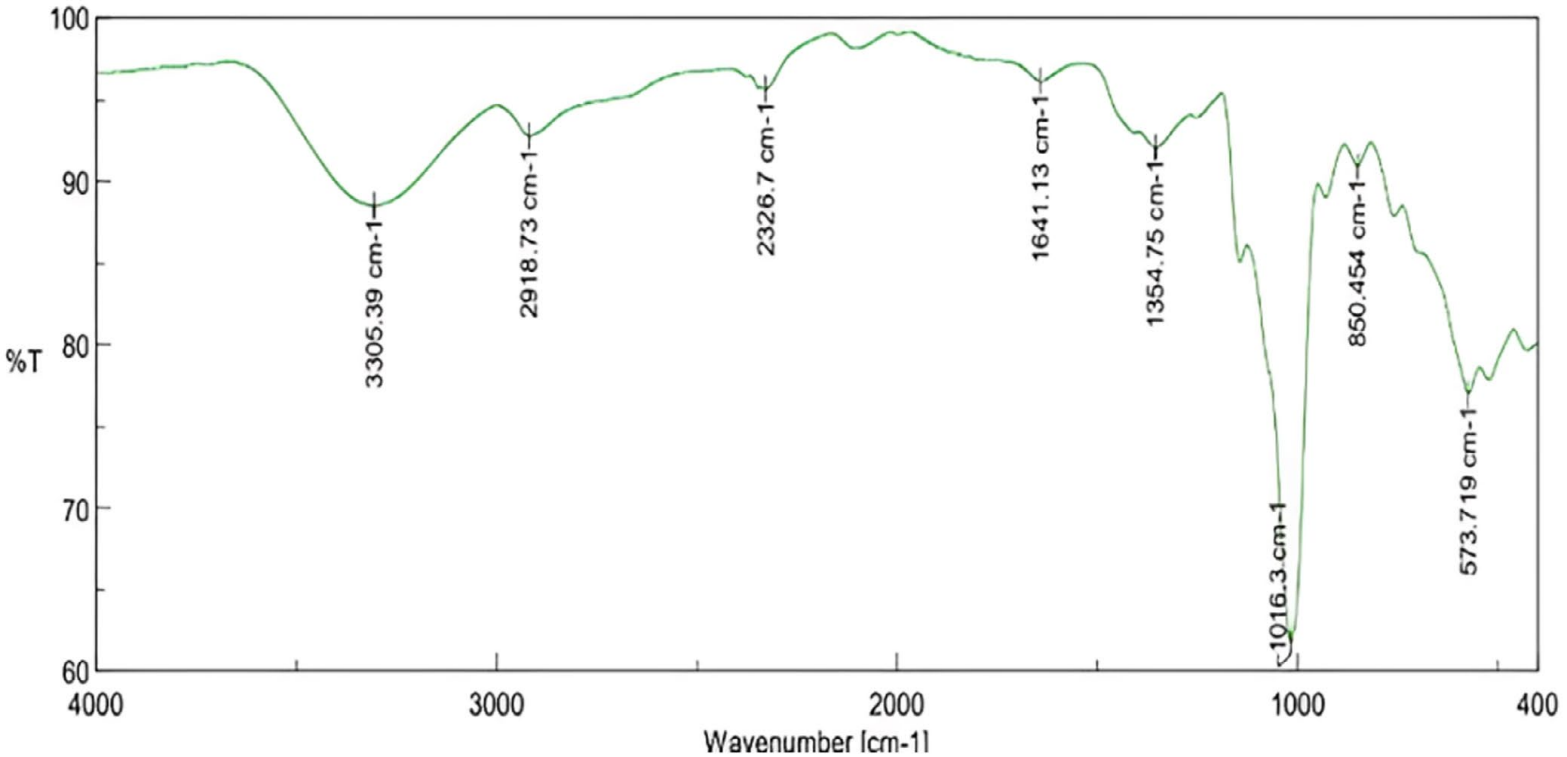
Fourier‐transform infrared (FTIR) spectrum of spray‐dried chlorophyll powder.

Fourier‐transform infrared (FTIR) spectroscopy was employed to identify characteristic functional groups and assess the structural integrity of chlorophyll after spray drying (Figure 5).

The FTIR spectrum displayed a broad absorption band at approximately 3305 cm^−1^, corresponding to O–H stretching vibrations. A peak at 2918 cm^−1^ was assigned to C–H stretching vibrations of aliphatic chains. The absorption around 2326 cm^−1^ was associated with N–H‐related vibrations and structural features of the pyrrolic rings in the porphyrin macrocycle. A distinct band at 1641 cm^−1^ corresponded to C=O stretching vibrations, while the peak at 1354 cm^−1^ was attributed to C–N and/or C–O vibrations. The strong band at 1016 cm^−1^ was characteristic of C–O–C stretching, typically associated with ether linkages in carbohydrate‐based wall materials. The preservation of these characteristic absorption bands indicates that the principal functional groups of chlorophyll remained intact after spray drying. Notably, no new absorption bands were detected that would suggest degradation of the tetrapyrrole ring system. Furthermore, no prominent absorption band was detected in the 1730–1700 cm^−1^ region, which is often associated with carbonyl groups of chlorophyll degradation products. However, because FTIR cannot reliably differentiate chlorophyll from its Mg‐free derivative pheophytin, this observation is only suggestive and does not, by itself, confirm that demetallation of Mg^2+^ was prevented (Li et al. 2018).

Comparative analysis with previously reported FTIR spectra of chlorophyll extracted using NADES systems revealed similar absorption features, including broad O–H stretching (~3300 cm^−1^), C–H stretching (2940–2924 cm^−1^), and C=O stretching (1641–1646 cm^−1^). Minor peak shifts (±10–15 cm^−1^) may be attributed to differences in sample matrix composition and hydrogen‐bond interactions between chlorophyll and maltodextrin in the encapsulated powder (Rumiyati et al. 2024). Overall, the FTIR results indicate that the principal functional groups and the overall molecular framework of chlorophyll were preserved after spray‐drying. However, because FTIR cannot reliably distinguish chlorophyll from its Mg‐free derivative, pheophytin, this observation should not be taken as direct evidence for the absence of pheophytinisation. It is therefore interpreted in conjunction with UV–Vis, HPLC, and colourimetric data, which provide more specific and sensitive indicators of chlorophyll degradation and pheophytin formation in subsequent studies.

#### 3.3.3 Solid‐State Properties by XRD

**FIGURE 6:**
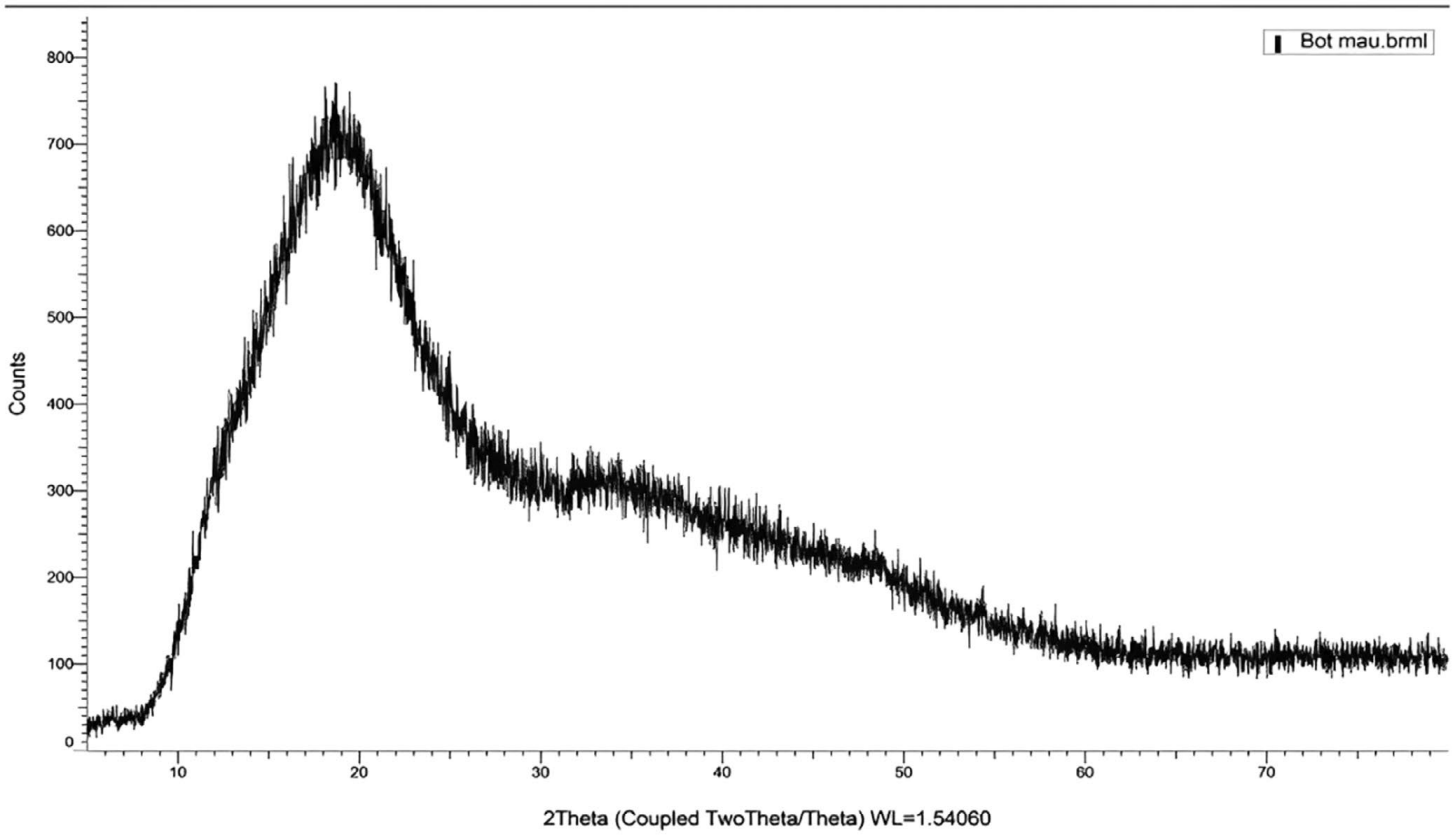
X‐ray diffraction (XRD) pattern of spray‐dried chlorophyll powder.

X‐ray diffraction (XRD) analysis was conducted to determine the solid‐state structure and crystallinity of the spray‐dried chlorophyll powder (Figure 6).

The diffraction pattern exhibited a broad halo over the 2θ range of approximately 15°–25°, without sharp, well‐defined diffraction peaks, indicating that the spray‐dried powder was predominantly amorphous and lacked long‐range crystalline order. Rapid solvent evaporation during spray drying likely restricted molecular rearrangement and crystal formation, favoring the formation of an amorphous matrix. Such a structure may facilitate powder dissolution and reconstitution; however, amorphous materials can undergo structural relaxation, stickiness, caking, or recrystallisation when exposed to elevated temperature or humidity. In the present study, the glass transition temperature (Tg) was not determined; therefore, the physical and storage stability of the amorphous matrix cannot be inferred from the XRD results alone. Further studies should determine Tg by differential scanning calorimetry at controlled moisture contents and evaluate stability under different temperature and relative‐humidity conditions. A similar amorphous halo in the 2θ region of 18°–25° has been reported for spray‐dried chlorophyll encapsulated with maltodextrin and whey protein isolate, supporting the predominance of an amorphous phase in this type of system (Ledari et al. 2024).

Overall, the FTIR spectrum indicated that the principal functional groups associated with the chlorophyll–maltodextrin matrix were preserved after spray drying. However, FTIR cannot reliably distinguish chlorophyll from its Mg‐free derivatives or provide compound‐specific quantification. Therefore, these results should not be interpreted as direct evidence for the absence of pheophytinisation. Further analysis using HPLC‐DAD and/or LC–MS/MS with authentic chlorophyll and pheophytin standards is required to confirm pigment composition and degradation.

#### 3.3.4 Physicochemical Parameters

The physicochemical properties of the spray‐dried chlorophyll powder were evaluated after the powdered formulation was obtained. The results of these analyses are summarized in Table 3.

**TABLE 3:** Physicochemical parameters of spray‐dried chlorophyll powder.

No.	Parameter	Unit	Results
1	Moisture content	%	3.25 ± 0.11
2	Bulk density	g/cm^3^	0.37 ± 0.007
3	Particle density	g/cm^3^	2.58 ± 0.15
4	Tapped density	g/cm^3^	0.48 ± 0.007
5	Flowability	CI (%)	23.45 ± 2.59
		HR	1.30 ± 0.02
6	Porosity	%	81.40 ± 1.80
7	Solubility	%	83.72 ± 1.11

The spray‐dried chlorophyll powder exhibited a moisture content of 3.25% ± 0.11%, bulk density of 0.37 ± 0.007 g/cm^3^, particle density of 2.58 ± 0.15 g/cm^3^, and tapped density of 0.48 ± 0.007 g/cm^3^ (Table 3). The low moisture content indicates effective drying and suitability for extended storage. The moderate bulk density reflects limited interparticle void space, potentially reducing oxygen penetration into the powder matrix. In comparison with the study of Femat et al., spray‐dried chlorophyll encapsulated with maltodextrin and fructan exhibited moisture contents of 3%–4% and bulk densities of approximately 0.35–0.40 g/cm^3^, indicating that the drying conditions were sufficient to promote uniform particle formation, limited internal voids, and rapid surface dehydration (Femat‐Castañeda et al. 2019). The present results are within a comparable range, suggesting adequate crust formation and structural stabilization during drying. Powder flowability is a critical parameter for packaging and downstream handling. The measured Carr index of 23.45% and Hausner ratio of 1.30 indicate fair flowability, implying that the powder can be handled and blended with acceptable performance. This behavior can be explained by the relationship between bulk density and interparticle interactions: uniformly encapsulated particles with reduced surface stickiness exhibit lower cohesive forces and improved mobility. Nevertheless, strict control of storage conditions is required to prevent moisture uptake, which may impair flow performance. Similarly, spray‐dried orange powder formulated with maltodextrin (CI ≈22.8%, HR ≈1.29), reflecting moderate flowability associated with relatively spherical and smooth particle morphology (Sidlagatta et al. 2020).

The high porosity (81.40%) and solubility (83.72%) indicate good water penetration and reconstitution of the spray‐dried powder, supporting its potential use in dispersed food systems such as powdered beverages, dairy products, and sauces. Rapid crust formation during spray drying may generate hollow or porous particles that facilitate wetting and dispersion. Similar solubility values (> 80%) have been reported for hollow, wrinkled particles in spray‐dried pigment powders (Kang et al. 2019). However, suitability depends on the specific food matrix and requires further assessment of dispersibility, sedimentation, and color stability. Direct comparison with commercial water‐soluble chlorophyll powders is limited because many contain chlorophyllin derivatives, whereas the present formulation comprises maltodextrin‐encapsulated natural chlorophyll, which is intrinsically lipophilic.

#### 3.3.5 Microbiological Analysis, Heavy Metal Content, and Residual Solvent

The results of microbiological analysis, heavy metal content, and residual solvent levels are presented in Table 4.

**TABLE 4:** Microbiological analysis, heavy metal content, and residual solvent levels of chlorophyll powder.

No.	Criteria	Results	Unit	Methods
1	Total aerobic mesophilic count	1.4 × 10^4^	CFU/g	TCVN 4884–1:2015 (ISO 4833‐1:2013)
2	Total yeast and mold	3.0 × 10^1^	CFU/g	TCVN 8275–2:2010 (ISO 21527‐2:2008)
3	Arsenic (As)	90.00	μg/kg	AOAC 2015.01
4	Lead (Pb)	2398.89	μg/kg	AOAC 2015.01
5	Cadmium (Cd)	Not detected	μg/kg	AOAC 2015.01
6	Mercury (Hg)	Not detected	μg/kg	AOAC 2015.01
7	n‐hexane	Not detected	mg/kg	FST‐WI06 chapter 44A (2020) (HS‐GC/MS)

Microbiological analysis was performed to assess product safety and storage stability. The total aerobic plate count was 1.4 × 10^4^ CFU/g, while total yeast and mold counts were 3.0 × 10^1^ CFU/g (Table 4). These counts were within the acceptable microbiological limits for dried food powders and met the criteria recommended by the International Commission on Microbiological Specifications for Foods (ICMSF 2011), which specify 10^3^ and 10^4^ CFU/g for total aerobic microorganisms, and 10 and 10^2^ CFU/g for yeasts and molds, respectively. The low microbial load can be attributed to the reduced water activity achieved during spray drying, which inhibits microbial proliferation. Maintaining low microbial contamination is particularly critical for pigment powders, as microbial enzymes may catalyze chlorophyll degradation, leading to browning and reduced color intensity during storage. The concentrations of arsenic (As) and lead (Pb) were 90.00 μg/kg and 2398.89 μg/kg, respectively (Table 4). These levels are below the maximum limits specified in QCVN 4–10:2010/BYT (As ≤ 3.0 mg/kg; Pb ≤ 5.0 mg/kg for food color powders), confirming compliance with Vietnam's national food safety standards. These results indicate that the heavy metal contents of the spray‐dried chlorophyll powder were within the permissible limits of the applicable food safety standards. Residual n‐hexane was analyzed using GC–MS, and the compound was not detected, with a method detection limit of 10.0 mg/kg. This value is well below the regulatory limit of 50.0 mg/kg stipulated for food applications. The absence of detectable solvent residues confirms the effectiveness of the evaporation and drying stages and supports the chemical safety of the final product.

Overall, the combined morphological, structural, physicochemical, microbiological, and safety analyses demonstrate that the spray‐dried chlorophyll powder maintains molecular integrity, predominantly an amorphous structure, satisfactory functional properties, and compliance with food safety standards.

## 4 Conclusion

An integrated microwave‐assisted extraction and spray‐drying process was developed for the recovery of chlorophyll from *C. submersum* . RSM identified microwave power and extraction time as the principal linear factors affecting total chlorophyll content, while the significant quadratic effect of MgCO_3_ indicated an optimal concentration within the investigated range. Preferred spray‐drying conditions were identified as 25% maltodextrin, a feed rate of 6 mL/min, and an inlet‐air temperature of 160° *C. SEM* showed the formation of predominantly spherical, wrinkled particles; FTIR revealed spectral features consistent with the chlorophyll–maltodextrin matrix but could not specifically distinguish chlorophyll from its degradation products; and XRD indicated a predominantly amorphous structure. The resulting powder exhibited high solubility, acceptable flowability, and compliance with the evaluated microbiological, heavy‐metal, and residual‐solvent criteria. These findings demonstrate the potential of *C. submersum* as an underutilized source of natural green pigment and provide a laboratory‐scale basis for further process development. Additional studies using HPLC‐DAD and/or LC–MS/MS with authentic standards are required to quantify individual chlorophylls and their degradation products. Storage‐stability studies, food‐matrix application trials, and pilot‐scale validation are also needed before industrial application.

## Author Contributions

**Hoang Thi Ngoc Nhon:** conceptualization, methodology, validation, writing – original draft, visualization, formal analysis, data curation, software, resources. **Lam Nguyen Dan Khoa:** formal analysis. **Le Thi Hong Anh:** conceptualization, writing – review and editing, visualization, validation, methodology, project administration, supervision, software.

## Conflicts of Interest

The authors declare no conflicts of interest.

## Data Availability

The data that support the findings of this study are available from the corresponding author upon reasonable request.
